# Genomic interplay in bacterial communities: implications for growth promoting practices in animal husbandry

**DOI:** 10.3389/fmicb.2014.00394

**Published:** 2014-08-12

**Authors:** Piklu Roy Chowdhury, Jessica McKinnon, Ethan Wyrsch, Jeffrey M. Hammond, Ian G. Charles, Steven P. Djordjevic

**Affiliations:** ^1^The ithree institute, University of Technology SydneySydney, NSW, Australia; ^2^NSW Department of Primary Industries, Elizabeth Macarthur Agricultural InstituteCamden, NSW, Australia

**Keywords:** multi drug resistance, lateral gene transfer, bacterial genomes, complex resistance loci, Antimicrobial growth promotion

## Abstract

The discovery of antibiotics heralded the start of a “Golden Age” in the history of medicine. Over the years, the use of antibiotics extended beyond medical practice into animal husbandry, aquaculture and agriculture. Now, however, we face the worldwide threat of diseases caused by pathogenic bacteria that are resistant to all existing major classes of antibiotic, reflecting the possibility of an end to the antibiotic era. The seriousness of the threat is underscored by the severely limited production of new classes of antibiotics. Evolution of bacteria resistant to multiple antibiotics results from the inherent genetic capability that bacteria have to adapt rapidly to changing environmental conditions. Consequently, under antibiotic selection pressures, bacteria have acquired resistance to all classes of antibiotics, sometimes very shortly after their introduction. Arguably, the evolution and rapid dissemination of multiple drug resistant genes *en-masse* across microbial pathogens is one of the most serious threats to human health. In this context, effective surveillance strategies to track the development of resistance to multiple antibiotics are vital to managing global infection control. These surveillance strategies are necessary for not only human health but also for animal health, aquaculture and plant production. Shortfalls in the present surveillance strategies need to be identified. Raising awareness of the genetic events that promote co-selection of resistance to multiple antimicrobials is an important prerequisite to the design and implementation of molecular surveillance strategies. In this review we will discuss how lateral gene transfer (LGT), driven by the use of low-dose antibiotics in animal husbandry, has likely played a significant role in the evolution of multiple drug resistance (MDR) in Gram-negative bacteria and has complicated molecular surveillance strategies adopted for predicting imminent resistance threats.

## Introduction

Antibiotics are central to modern medical and veterinary practice. However, the effectiveness of existing antibiotics in both medical and veterinary practice is under threat due to the rapid rise of multiple drug resistance (MDR) (Gootz, 2010). The 2014 World Health Organisation (WHO) Global report on surveillance of antimicrobial resistance makes this point clear—“A post-antibiotic era—in which common infections and minor injuries can kill—far from being an apocalyptic fantasy, is instead a very real possibility for the 21st century.” (WHO, 2014). This possibility was illustrated by antibiotic treatment failure in the 2011 epidemic in Germany in which enteroaggregative *Escherichia coli* (EAHEC) O104:H4 caused a large outbreak of acute gastroenteritis and haemolytic uremic syndrome. EAHEC O104:H4 is an example of a newly emerged pathogen that possesses novel combinations of acquired antibiotic resistance genes and virulence factors. In the case of EAHEC O104:H4 an enteroaggregative *E. coli* had acquired a *stx*_2_ shiga toxin gene, a characteristic feature of enterohaemorrhagic *E. coli* genomes, via a bacteriophage (Grad et al., 2013); a plasmid encoded CTX-M-15 gene (encoding the antibiotic resistance factor, extended-spectrum beta-lactamase) (Kunne et al., 2012); and a chromosomally-located complex antibiotic resistance gene cluster, identified recently as the region of divergence 1 (Frank et al., 2011; Januszkiewicz et al., 2011; Ahmed et al., 2012; Radosavljevic et al., 2014). The extensively antibiotic resistant *E. coli* clonal group ST131 is similarly a globally emergent pathogen with novel combinations of acquired antibiotic resistance genes and virulence factors (Johnson et al., 2010; Andersen et al., 2013; Banerjee and Johnson, 2013; Price et al., 2013). Since 2011 there has been an increase in the number of reports describing hybrid *E. coli* genomes with a repertoire of virulence and resistance genes, complicating precise pathotype allocations (Bielaszewska et al., 2014; Prager et al., 2014; Toval et al., 2014). However, it is difficult to determine whether the increased number of reports equates to an increasing rate in the emergence of these novel pathogens. It could simply mean that the increase in reports of novel pathogens is due to the growing affordability of, and improvements in, whole genome sequencing technologies and molecular diagnostics that enable rapid and accurate identification of emerging clones. However, increasing emergence of novel strains is also likely- given the rapid rise in human population, increased demands on food production and the speed by which people and commodities move globally. Either way, the suite of acquired resistance genes these novel pathogens harbor clearly indicates the significant role played by Lateral Gene Transfer (LGT) in shaping these genomes. Antibiotic resistance genes have a propensity to cluster on mobile elements and consequently a single LGT event can result in resistance to multiple antibiotic classes (Toleman and Walsh, 2011). Notably also, these trends are not restricted to Gram-negative pathogens but apply additionally to Gram-positive genomes.

Antibiotic resistance existed in nature long before the discovery of antibiotics 70 years ago. For example, genes encoding resistance to β–lactam(s), tetracycline and glycopeptide antibiotics have been identified from ancient metagenomic samples of 30,000 years old Beringian permafrost sediments (D'Costa et al., 2011). Controlling the spread of antibiotic resistance into diverse pathogens, and particularly multidrug resistance, requires better use of currently available antibiotics. It is insufficient to rely on the development of new antibiotics or antibiotic adjuncts. More effective use of existing antibiotics can be guided by molecular surveillance of microbiomes.

It is now clear that multiple distinct antibiotic resistance genes forming complex resistance gene loci (CRL) in MDR bacterial genomes were likely acquired from disparate backgrounds. Many MDR bacteria resulting in hospital infections carry genes encoding resistance to antibiotics used frequently in food animal production or resistance genes that have been acquired from aquatic and soil environments (Szczepanowski et al., 2009; Popowska and Krawczyk-Balska, 2013; Wibberg et al., 2013). This is not surprising given that New Generation DNA sequencing has revealed a vast repertoire of drug resistance determinants across diverse microbiomes (Forsberg et al., 2012). It has, however, largely gone unnoticed, because of the way in which molecular surveillance is carried out differentially in hospital and veterinary microbiology laboratories.

Screening procedures in hospital and veterinary laboratories are quite justifiably influenced by the urgent need to deal with an infectious threat; thus screening is carried out on the basis of resistance to the set of antibiotics that are of relevance to the respective unit. This means, for example, that although mercury resistance transposons are one of the largest carriers of multiple antibiotic resistance genes, veterinary and clinical hospital microbiology laboratories do not screen for mercury resistance. Focused surveillance approaches have thus resulted in an underestimated view of the real problem of antibiotic resistance. In recognition of the importance of fully understanding the problem of antibiotic resistance, recent WHO and UK government reports have advocated a “one health” approach that uses genomic surveillance of both clinical and food animal isolates (Department of Heath and Department for Environment Food and Rural Affairs, 2013; WHO, 2014).

The disparate reservoirs of drug resistance determinants raises important questions regarding the extent of transmissibility of the pool of resistance genes and the range of bacteria that can acquire them as a consequence of LGT. This review will give an overview of antibiotic resistance gene reservoirs with particular focus on the reservoirs in food animals, soil and water resulting from the in-feed use of large quantities of sub-therapeutic doses of antibiotics as growth promoting agents in food animal husbandry. It will highlight a significant gap in our knowledge regarding the evolution of MDR in bacteria: the range of factors that trigger the selection and mobilization of antibiotic-resistance genes from environmental reservoirs into clinically-relevant organisms. The complexity of the evolution of MDR in bacteria has myriad implications, including, for example, for the choice of alternative food animal growth promoters other than in-feed antibiotics. This review will point to considerations relevant to this choice.

## An assessment of the repertoire of resistance genes and effects of low dose antibiotics

### Antibiotic resistance genes present in disparate reservoirs

The antibiotic resistance genes that accumulate in the genomes of bacteria are likely to have their origins in disparate reservoirs, a conclusion arrived at in several independent recent studies on the evolution of MDR (Aminov, 2011; Amos et al., 2014; Guerra et al., 2014; Hsu et al., 2014; Ojer-Usoz et al., 2014) and evidenced also by the widespread dissemination of various antibiotic resistance genes, including those encoding resistance to extended spectrum β-lactamases in birds and other wildlife species (Costa et al., 2008; Poeta et al., 2008; Simoes et al., 2010). Aquatic and soil environments serve as sinks of older generation antibiotics including sulphonamides, tetracycline, amoxicillin, ampicillin and trimethoprim (Boxall, 2004; Monteiro and Boxall, 2009). The pools of antibiotic resistance genes available for dissemination (known as the “resistome”) are enriched by veterinary and agricultural practices, human sewage and hospital waste (Tennstedt et al., 2005; Galvin et al., 2010; Heuer et al., 2011; Wellington et al., 2013; Hsu et al., 2014; Ojer-Usoz et al., 2014).

Genes imparting antibiotic resistance move within bacterial population(s) through LGT, independent of the linear “parent to progeny” descent (Tennstedt et al., 2005; Stokes and Gillings, 2011). The genes can be from a wide “gene pool” and can be transmitted between closely and distantly related species. Examples in which LGT plays a significant role in the evolution of important multi-resistant pathogenic bacterial genomes include, but are not restricted to, *E. coli, Pseudomonas aeruginosa*, and *Vibrio cholerae*. Notably, these organisms can survive in a range of environmental niches outside of the human body, including fomites in hospitals, soil and aquatic environments. Consequently, there exists diverse niche-adapted pools of microbial communities that can provide opportunities for the sampling and exchange of genetic information. Further, these opportunities are likely to be influenced significantly by the ecology of the niche-adapted microbial communities (Heuer et al., 2009, 2011; Galvin et al., 2010; Forsberg et al., 2012; Wellington et al., 2013).

### Food animals, soil, and water are important reservoirs of antibiotic resistance genes

Reservoirs of antibiotic resistance genes that are increasingly recognized as important are the gastrointestinal tract of food animals, soil and water. This is because of the practice of using sub-therapeutic doses of antibiotics to promote the growth of food animals and the prophylactic use of antibiotics to prevent disease in food animals. A significant percentage of administered antibiotics are excreted in animal waste. Un-metabolized antibiotics concentrate in animal waste ponds and, depending on their chemical composition, partition to different fractions during the wastewater treatment processes (Kolpin et al., 2002; Ghosh and Lapara, 2007; Watanabe et al., 2010; Cheng et al., 2013; Li et al., 2013). Thus, antibiotics enter into environments/ecosystems where bacteria pathogenic to humans may also survive (Sarmah et al., 2006). The antibiotics used with food animals for growth promotion and prophylactically include a set of FDA (FDA, 2011) approved drugs, recognized by the World Health Organization as important to treat human diseases (Table 1) (Aarestrup et al., 2008). Further, the quantities of antibiotics administered to food animals are very large. In Australia, in the period 2005 to 2010, 98% of veterinary antibiotics sold were for use in food animals. Only approximately 43% were sold for therapeutic or prophylactic purposes (no distinction could be drawn between “therapeutic” and “prophylactic” uses as it was difficult for those surveyed to estimate the proportions of products used for these purposes). This means that, on average, for the surveyed period, sales of antimicrobials for growth promotion averaged 35.3 tons; in 2005–2006 this was as much as 47.2 tons (APVMA, 2014). Importantly, a large proportion of the antimicrobials sold (almost 77%), were administered in feed.

**Table 1:** **List of FDA approved Antibiotic classes and countries still using them in growth promotion, associated resistance genes and genetic scaffolds that laterally co-mobilize them with other antibiotic and metal resistance genes**.

**Antibiotic classes: specific examples**	**Countries still using them**	**Associated resistance genes**	**Association of genes with mobile elements**	**Examples of co-resistances**
Penicillins: amoxicillin, ampicillin	United States (Mathers et al., 2011), Sudan (Eltayb et al., 2012)	blaTEM genes	blaTEM genes transposons (Bailey et al., 2011) and plasmids (Cain et al., 2010)	Lead, cadmium, zinc and chromium (Yamina et al., 2012) *mer* genes;
				mercury (Mcintosh et al., 2008)
Glycopeptides: avoparcin, vancomycin	Mexico (Maron et al., 2013)	*van* genes	*van genes* [transposons (Jensen et al., 1999; Leavis et al., 2003) and plasmids (Zhu et al., 2010, 2013)]	*tcrB* gene; copper (Hasman and Aarestrup, 2002)
Macrolides: erythromycin, tylosin, tilmicosin, kitasamycin, oleandomycin	United States (Kim et al., 2012), Australia (Hughes and Heritage, 2004), Mexico (Maron et al., 2013)	*erm* gene cluster	*erm* gene cluster [transposons (Li et al., 2011; Ramos et al., 2012) and plasmids (Wendlandt et al., 2014)]	*erm* genes; tetracycline and streptogramin (Ramos et al., 2012)
		CmeABC multi-drug efflux pump (Lin et al., 2007)		*tcrB* gene; copper (Hasman and Aarestrup, 2002)
Streptogramins: virginiamycin, quinupristin-dalfopristin	United States (Kieke et al., 2006), Australia (Hughes and Heritage, 2004), Mexico (Maron et al., 2013)	*vatD* and *vatE*	*vatD* and *vatE* plasmids (Allignet and El Solh, 1999)	*erm* genes; macrolide and tetracycline (Ramos et al., 2012)
		*erm* gene cluster	*erm* gene cluster [transposons (Li et al., 2011; Ramos et al., 2012) and plasmids (Wendlandt et al., 2014)]	
		*satA* (Hammerum et al., 1998)		
		*varS* (Lee et al., 1999; Kieke et al., 2006)		
Sulfonamides: sulfisoxazole, sulfadimethoxine, sulfamethazine	Sudan (Eltayb et al., 2012), United States (APUA, 2010)	*sul* genes	*sul* genes [transposons (Cain et al., 2010), plasmids (Wu et al., 2010) and clinical class 1 integrons (Stokes and Hall, 1989; Wu et al., 2010)]	*czcA* gene; zinc, cadmium and cobalt (Stokes et al., 2006; Gillings et al., 2008) *mer* genes; mercury (Mcintosh et al., 2008)
Tetracyclines: chlortetracycline, oxytetracycline, doxycycline	United States (Cox and Popken, 2010; Mathers et al., 2011), China (Wu et al., 2010), Sudan (Eltayb et al., 2012)	*tet* genes	*tet* genes [transposons (Schmitt et al., 1979; Ramos et al., 2012) and plasmids (Han et al., 2012; Wendlandt et al., 2014)]	*erm* genes; macrolide and streptogramin (Ramos et al., 2012)
				*mer* genes; mercury (Mcintosh et al., 2008)
Polypeptides: bacitracin	Mexico (Maron et al., 2013)	*rgpA-F, mbrA-D* (Tsuda et al., 2002)	*bcr* [plasmids (Tremblay and Archambault, 2013)]	
		*bcr*		
Amphenicols: chloramphenicol	China (Li et al., 2013)	*cmlA*	*cat* gene [transposons, (Cain and Hall, 2012), plasmids (Mcintosh et al., 2008) and integrons (Bunny et al., 1995)]	*mer* genes; mercury (Mcintosh et al., 2008)
		*floR*		
		*fexA and fexB*		
		*cfr*		
		*cat* gene		

The practice of using sub-therapeutic doses of antibiotics to promote growth of food animals became widespread following initial studies of the effect of antibiotics on the growth of broiler chickens (Elam et al., 1953; Jacobs et al., 1953; Izat et al., 1990; Dibner and Richards, 2005; Castanon, 2007). Antibiotics are used for growth promotion in doses lower than the recommended minimum effective concentrations (MEC) for therapeutic purposes but often at concentrations greater than the minimum inhibitory concentration (MIC) of specific drug/microbe combinations (Berrang et al., 2007; Alexander et al., 2008; Mirzaagha et al., 2011; Holman and Chenier, 2013). The practice of using antibiotics to promote growth of food animals has been banned in several countries in Europe due to concerns over the development and spread of antimicrobial resistance (Gilbert, 2011; Maron et al., 2013).

### Impact of sub-inhibitory concentrations of antibiotics on bacteria

The practice raises concerns over the development and spread of antimicrobial resistance because sub-inhibitory concentrations of antibiotics can enhance LGT (Barr et al., 1986; Torres et al., 1991; Stevens et al., 1993); potentially act as signal transduction molecules (Romero et al., 2011) in the transition from planktonic to biofilm phase during the process of monomicrobial and polymicrobial biofilm formations,(Bagge et al., 2004; Hoffman et al., 2005); and globally modulate transcriptional activity. Sub-therapeutic doses of β-lactam antibiotics has been shown to enhance the rate of conjugative plasmid transfer (Barr et al., 1986) and tetracycline has been implicated in driving the lateral transfer of integrative conjugative elements (Torres et al., 1991; Stevens et al., 1993). Conjugative transfer of single-stranded (ss) DNA (the mechanism by which plasmids are transferred from donor to recipient cells) induces the bacterial SOS response, which in turn up-regulates expression of the integron integrase resulting in the capture or exchange of resistance genes in Gram-negative pathogens (Baharoglu et al., 2010). Integron-mediated gene shuffling events in *V. cholerae* and *E. coli* were elevated significantly (4.5-fold and 37-fold increase respectively) in response to antibiotic mediated stress, which in turn triggered the SOS response. In addition, the study also reported up-regulation of the frequency of integron-associated excision/shuffling of gene-cassettes by 141-fold in *V. cholera* and 340-fold in *E. coli*, in a SOS response mediated event. In a recent study, Zhu et al. (2013) demonstrated the presence and relative abundance (an increase of 198-fold on average) of 149 antibiotic resistance genes (of 244 included in the study) conferring resistance to common in-feed and veterinary antibiotics used in the swine farming industry in China within 36 metagenomic samples extracted from compost, manure and soil. The study also provided evidence of abundance and enrichment of common transposase genes [identified in the course of a previous study (Aziz et al., 2010)] known to be frequently associated with antibiotic resistance genes. The conclusions of the study included a statement of the likely roles played by the subset of mobile elements in LGT of resistance genes in China (Zhu et al., 2013).

In a pioneering transcriptome analyses study, Goh et al. provided direct evidence of the global modulation of transcriptional activity of genes in *Salmonella enterica* serovar Typhimurium in the presence of sub-inhibitory concentrations of antibiotics like erythromycin (used as Antimicrobial growth promoters or AGP for poultry and swine) and rifampicin (Goh et al., 2002). Further gene-expression studies have now presented data on the influence of sub-inhibitory concentrations of antibiotics on the global transcriptome of genes related to virulence (Subrt et al., 2011), colonization (Bagge et al., 2004), motility, SOS stress response (inducible DNA repair system) and biofilm formation (Kaplan et al., 2012) for many important human pathogens (Davies et al., 2006). Notably, stress responses have been shown to enhance bacterial adaptation through increased mutation and LGT (Aertsen and Michiels, 2006; Baharoglu and Mazel, 2011).

## LGT of resistance genes and co-selection of other resistance determinants by the formation of complex resistance loci

### Complex resistance gene loci—multiple antibiotic resistance genes and mobile elements

Genes that encode resistance to antibiotics often appear clustered in the genomes of many bacteria, forming CRL (Parkhill et al., 2001; Szczepanowski et al., 2005, 2009; Tennstedt et al., 2005; Roy Chowdhury et al., 2009, 2011; Venturini et al., 2010; Toleman and Walsh, 2011). In addition to antibiotic resistance genes, the CRL possess diverse mobile genetic elements (predominantly insertion elements, transposons and integrons) clustered either in independently replicating units, like plasmids, or in genomic islands within the bacterial chromosome. Mobile elements facilitate dissemination of CRL within bacteria. Acquisition of a CRL by any bacterium thus provides a survival mechanism to it when exposed to an entire range of antimicrobial agents. CRL evolve rapidly, especially within Gram-negative bacteria, and they generally contain hotspots where additional resistance genes can accumulate. Lateral transfer of genes within the gastrointestinal tracts of humans and food animals, and in reservoirs where environmental bacteria mix with bacteria derived from anthropogenic activities (sewage, hospital waste and food production animal waste) significantly contribute to these evolutionary processes (Forsberg et al., 2012). Although mobile genetic elements are a key component of CRL, there is currently limited understanding of the different types of mobile genetic element(s) that may contribute to: (1) the spread of CRL; (2) the diversity of resistance gene reservoirs that can be utilized by the mobile elements to form CRL and (3) the mechanisms by which resistance genes assemble on laterally mobile segments of DNA.

### Integrons are mobile elements associated with CRL and found in diverse bacteria

Integrons represent a group of genetic elements that are most commonly found in association with CRL and have been implicated in the rapid evolution of multi-drug resistance within Gram-negative pathogens (Martinez et al., 2013). Based on the amino acid sequences of the integrase/recombinase gene *intI* (Recchia and Hall, 1995) integrons can be differentiated into several classes, all of which are found in diverse microbial communities (Marquez et al., 2008). Classes 1–3 are most frequently associated with the dissemination of MDR within Gram-negative bacteria, although recently class 1 integrons have also been identified in Gram-positive bacteria (Nandi et al., 2004; Shi et al., 2006; Xu et al., 2010, 2011). Exchange of resistance genes within different classes of integrons (from diverse bacteria) has been reported in a range of microbial communities (Labbate et al., 2008; Roy Chowdhury et al., 2009). The role of class 1 integrons in the evolution of MDR within hospitals is most comprehensively documented (Djordjevic et al., 2013; Martinez et al., 2013), although they are also frequently reported from environmental bacteria (Holmes et al., 2003; Stokes et al., 2006). Functionally, a class 1 integron is a two-component site-specific gene recombination system. Structurally a class 1 integron comprises an integrase gene and a tandem array of a variable number of independently acquired mobile genes. The independently mobile units of a class 1 integron are called “gene cassettes” and are made up of a promoter-less open reading frame and a recombination site. Class 1 integrons most frequently contain multiple gene cassettes, in structures called “cassette arrays,” that can be expressed from a single promoter (Stokes and Hall, 1989) in an operon-like manner. Exchange/insertion/deletion of gene cassettes (by the process of site-specific recombination) and expression of these genes enables their host to exhibit multiple “acquired” resistance phenotypes simultaneously and relatively quickly (on an evolutionary time scale).

A key component of the integron integrase is the integrase promoter (P_int_) (Stokes and Hall, 1989). The regulation and expression of the integron integrase is an adaptive response to stress, including that induced by sub-lethal dosages of antibiotics in any environment (Guerin et al., 2009). Hocquet et al. described a metronidazole induced cassette rearrangement in a class 1 integron that resulted in the formation of a fused *gcuF1-bla*_OXA−28_ cassette, which subsequently led to the expression of the fused β-lactamase gene and ceftazidime resistance in a *P. aeruginosa* isolate (Hocquet et al., 2012). A LexA binding site is found conserved across the vast majority of integrase genes, indicating the likelihood of bacterial SOS mediated up-regulation of most integron integrases (Guerin et al., 2009). Importance of the LexA binding site has been experimentally demonstrated in *Vibrio* spp. and *E. coli* (Guerin et al., 2009).

Class 1 integrons isolated from clinical samples, are predominantly linked to the Tn*3* family of transposons, particularly Tn*21* (Liebert et al., 1999) and Tn*1696* (Partridge et al., 2001), which in turn harbor clustered mercury resistance genes. Class 1 integrons also have a propensity of targeting resolution sites of plasmids or other transposons on plasmid backbones (Kholodii et al., 1995; Minakhina et al., 1999). Thus, in addition to carrying genes conferring MDR, class 1 integrons also associate with other mobile elements, which independently carry other combinations of resistance genes to form laterally mobile resistance scaffolds (Parkhill et al., 2001; Szczepanowski et al., 2005; Tennstedt et al., 2005; Wibberg et al., 2013).

### Co-selection and transfer of antibiotic and other resistance genes—the role of plasmids

Plasmids play a central role in the lateral transfer of CRL within both closely and distantly related Gram-negative bacteria. Identical CRL are frequently seen piggy-backing on different plasmid backbones isolated from different hosts, supporting the role of plasmids in the transfer of drug resistance loci. Such events are best exemplified by plasmids carrying identical complex Tn*21*-associated MDR regions (Tennstedt et al., 2005; Szczepanowski et al., 2009; Venturini et al., 2013). Recent examples of such plasmids with complex resistance loci isolated from waste water treatment plants include IncF plasmids pRSB225 (Wibberg et al., 2013) and pRSB107 (Szczepanowski et al., 2005) and the series of IncP-1 plasmids reviewed recently by Popowska and Krawczyk-Balska (2013). Although isolated from waste water treatment plants, all these plasmids and a set of 140 plasmids described in a recent plasmid metagenomic study by Szczepanowski et al. clearly indicate that the resistance gene pool within the CRL on the plasmids are an admixture of genes that have originated from different bacterial communities (Szczepanowski et al., 2009).

The co-selection of antibiotic resistance genes by plasmids containing transposons with genes encoding resistance to heavy metals (such as mercury, cadmium, copper, and zinc) adds to the complexity of the evolution of laterally mobile clustered resistance regions (Seiler and Berendonk, 2012). This is particularly so when considering food animals and their environments (e.g. soil and water) as reservoirs of antibiotic resistance determinants; metals like copper and zinc, all of which are often used in animal husbandry for growth promotion (Jacob et al., 2010). This phenomenon of co-selection of resistance genes on plasmids increase the opportunity for microbial populations, that proliferate both in the gastrointestinal tracts of mammals and that survive in soil and aquatic environments, to act as major conduits in the flow of resistance genes between these reservoirs.

Plasmids pO26-CRL, pO26-CRL_125_ (isolated from an enterohemorrhagic *E. coli* specimen from a human with bloody diarrhea) and pO111-CRL_115_ (isolated from a bovine source in Australia) are examples of plasmids with identical clustered antibiotic and metal resistance genes that have been identified recently by our group (Venturini et al., 2010, 2013). The CRL in these plasmids are characterized by a Tn*21*-associated class 1 integron truncated by the presence of a composite IS*26* transposon, Tn*6026* (Cain et al., 2010). Composite transposons, another type of mobile element frequently associated with CRL, are defined as segments of DNA bounded by two related insertion elements (or IS elements). DNA segments between two IS elements mobilize intervening genes as a single unit. Composite transposons therefore have the ability to capture and mobilize random pieces of DNA from many diverse genetic locations, promoting genetic diversity. In relation to evolution of MDR, composite transposons can allow the movement of clustered antibiotic resistance genes from one bacterial genome to another through the capture of such genes into bacteriophages or conjugative plasmids that can then move readily between bacteria. Tn*6026* is an example of a composite transposon, which consists of two independently mobile composite transposons, Tn*6029* and Tn*4352* (Cain et al., 2010; Martinez et al., 2013). Collectively the CRL (described above as Tn*6026*) confers resistance to ampicillin, kanamycin, neomycin, streptomycin, sulfathiazole and trimethoprim. Similar structures, consisting of Tn*6029* derivatives and essentially consisting of the same group of resistance genes have been described in plasmids such as pASL01a, which circulates in commensal *E. coli* in the human population in West Africa (Labar et al., 2012), and pHCM1, from a human *E. coli* isolate from a patient living in the Mekong Delta in Vietnam (Parkhill et al., 2001). However, the group of plasmids described by us, pO26-CRL, pO26-CRL_125_, and pO111-CRL_115,_ is a clear example of plasmid mediated LGT of CRL between animal and human reservoirs in recent times.

IncA/C plasmids can provide conjugative functions in trans to mobilize integrative elements such as *Salmonella* Genomic Island 1 (SGI1) (Douard et al., 2010). SGI1 harbors genes encoding resistance to seven antibiotics (ampicillin, chloramphenicol, florphenicol, streptomycin, spectinomycin, tetracycline and sulphonamides). The resistance genes are clustered on a 13-kb class 1 integron, In104, embedded within a 42.4-kb genomic island embedded between *thdF* and *yidY* genes (Boyd et al., 2001; Levings et al., 2005). SGI1 is widely dispersed among a range of *S. enterica* serovars and *Proteus mirabilus* (Levings et al., 2005, 2006, 2007; Djordjevic et al., 2009; Le Hello et al., 2011, 2012), clearly indicating the lateral transfer of the element within bacteria occupying disparate hosts. A structurally similar CRL is also found in SGI2 described initially from a *S. enterica* serovar Emek strain in Australia. Major differences between SGI1 and SGI2 are in the physical location of the class 1 integron on the genomic backbone and in the different set of resistance gene cassettes seen associated with the integron found in SGI2. The integron, known as InEmek is a close variant of In104 seen in SGI1 (Levings et al., 2008) and the entire island shows characteristic features that mobilize SGI1.

## Novel strategies and alternatives to in-feed antibiotics

The growing knowledge of how LGT drives the evolution of MDR in bacteria and the importance of disparate pools of resistance genes has implications for the use of antibiotics as growth promoters in food animals, suggesting a need for novel alternatives to in-feed antibiotics. These alternatives should, in development, pay regard to: (1) the potential dangers for increasing evolution of MDR in bacteria when altering the microbial ecology of the gut (as most currently used growth promoters appear to do) (Dibner and Richards, 2005); and (2) consideration of questioning the effectiveness of in-feed antibiotics as growth promoters (Bengtsson and Wierup, 2006). Kim et al. conducted a comparative fecal microbiome study on two separate porcine populations, one of which was given a diet supplemented with the macrolide tylosin (a commonly used AGP), while the other group represented the control (Kim et al., 2012), i.e., did not receive tylosin, and reported the flux of microbial communities within them over a period of time. The fecal microbiome of each group was determined, which initially showed a prominent shift in microbial content for all species detected, proving that AGPs such as tylosin alter the native intestinal flora, resulting in less competition for nutrients and ultimately leading to the desired growth promoting effects. However, this study also showed that fecal microbiomes from the control group eventually became indistinguishable to those of the tylosin-fed group, offering an air of redundancy to the use of AGPs with respect to health and welfare of the animals. An important aspect of this study was that it was conducted in two separate farm settings, instead of the common choice among similar studies of an infectious disease isolation facility, making the results more comparable and applicable to the pig-farming industry.

Any alternative strategy for growth promotion in food animals over antibiotics needs to consider the growing understanding of the triggers for lateral transfer of CRL and the warning provided by evidenced movement of CRL between disparate bacterial hosts as consequences of administration of sub-inhibitory dosages of antibiotics. Controlling the spread of antibiotic resistance is not so simple as banning non-therapeutic in-feed antibiotics and using anything else as a growth promoter. This is clearly demonstrated by the problems associated with the use of heavy metals as growth promoters in relation to lateral co-transfer of associated antibiotic resistance genes. Further, an ideal alternative to in-feed AGPs should ideally meet the definition for feed additives set by The Official Journal of the European Union (Council Directive 89/107/EEC). The derivative clearly states “any substance not normally consumed as a food in itself and not normally used as a characteristic ingredient of food whether or not it has nutritive value, the intentional addition of which to food for a technological purpose in the manufacture, processing, preparation, treatment, packaging, transport or storage of such food results, or may be reasonably expected to result, in it or its by-products becoming directly or indirectly a component of such foods” (Council Directive 89/107/EEC). With respect to farming and animal husbandry, the novel additive should ideally improve the characteristics of the feed itself as well as dairy and deli products derived from the receiving animal. They should increase the efficiency of animal performance and welfare via the gastro-intestinal microbiota and satisfy the nutritional requirements of the animal. Additionally, the additive should not adversely impact the environment or human/animal health. From this perspective, it is questionable whether the environmental accumulation of minerals, such as zinc oxide (often used as an alternative to in-feed antibiotics), is worth the positive effects of growth promotion and prophylaxis for conditions such as *E. coli* mediated post-weaning diarrhea in piglets (Pluske, 2013).

A diverse range of options, which could possibly minimize or ideally alleviate forces that drive lateral transfer of resistance genes within the gut microbiome, have been proposed as alternatives of AGPs including, but not limited to, enzymes, nutraceuticals, amino acids and plant extracts (Askbrant et al., 1994; Hill et al., 2000; Barrerra et al., 2004; Schone et al., 2006; Muhl and Liebert, 2007; Nortey et al., 2007; Ragland et al., 2007; Jacela et al., 2008). These have shown varying outcomes in the promotion of growth in different animals (Supplementary Table 1). Therefore, it is most likely that a combination of these substitutes may serve as a better alternative that could account for all benefits and functions of in-feed antibiotics than any individual substance tested for the purpose. This idea was investigated within poultry by Ohimain and Ofongo (2012). They reported the effects of using a mixture of probiotics, prebiotics and enzymes as a dietary supplement in poultry. The probiotics and prebiotics had a synergistic effect, while the enzymes enhanced digestibility of the feed, increasing the amount of nutrients available for absorption. The combined effect improved digestive health in the chickens and consequently protected them against common microbial diseases such as enteritis (Ohimain and Ofongo, 2012). Probiotics, on the other hand, have been documented to prevent inflammation in the gut, reduce the incidence of meat contamination and promote growth, proving a propitious alternative to AGPs (Patterson and Burkholder, 2003). Use of non-pathogenic *E. coli* probiotics in combination with a low-protein diet in a porcine model was shown to reduce mucosal populations of pathogenic *E. coli* K88, subsequently decreasing the incidence of post-weaning diarrhea. These effects were attributed to the production of colicin by some bacteria introduced via the probiotic product (Bhandari et al., 2010) as well as the unavailability of protein normally utilized by the pathogenic *E. coli* for energy production. A similar study used a low-protein diet with the addition of essential amino acids (Heo et al., 2008) and described similar results. Further development of such strategies would assist in controlling the spread of antibiotic resistance, as well as reduce appearance of medical conditions such as post-weaning diarrhea in piglets, a major cause of economic hardship in swine production (Pluske, 2013).

One of the main problems of developing alternatives to in-feed AGPs is that the mechanism of action of AGPs is still largely unknown. No general consensus for the mechanism of action of AGP exists, however, several hypotheses have been proposed and evaluated, including: (1) reduction in total bacterial load as a consequence of administrated antibiotics; (2) reduction in the number of pathogenic bacteria resulting in better animal health; and (3) manipulation of the gut microflora in a way that natural immune and metabolic responses balance resulting in healthy animals (Dibner and Richards, 2005). Like existing alternatives to in-feed antibiotics, proposed novel alternatives tend to have a mechanism of action which directly affects the composition and quantity of bacteria within the animal's gastrointestinal system, suggesting a link between commensal bacteria and growth performance. The effects of AGP on porcine and broiler chicken intestinal microbiomes have been reported (Collier et al., 2003; Dumonceaux et al., 2006), as well as the positive effects of the change in composition of these microbiomes in response to in-feed antibiotics. These studies mirror those reported by Kim et al., as well as provide evidence to the hypothesis of this mode of action. Collier et al. and Dumonceaux et al. also identified bacterial composition within specific regions of the gastro-intestinal tract, and in doing so created a link between the alterations of bacterial communities most affected by the antibiotics and areas of the intestine most involved in nutrient absorption.

Several reports have linked the use of AGPs (Feighner and Dashkevicz, 1987; Knarreborg et al., 2004; Guban et al., 2006) to the reduction in activity of intestinal bile salt hydrolase (BSH). This enzyme directly affects lipid metabolism within the host and is produced by bacteria that occur naturally in the gut. The observation led to a new avenue of research into factors that target bacterial products rather than the bacteria themselves. Based strictly on an *in vitro* study, Wang et al. (2012) proposed the use of bile salt inhibitors as growth performance enhancers (Wang et al., 2012). Whether BSH inhibitors would adequately function in an *in vivo* model is still unclear. Zinc and copper, (common feed additives for increasing feed efficiency and promoting growth), were shown to cause inhibition of BSH and improve lipid metabolism. There are likely to be a number of non-metal BSH inhibitors, so this research path, with further studies into non-environmentally contaminating compounds, is a promising replacement for in-feed antibiotics.

## Concluding remarks

Laterally acquired DNA is a major driver of genome plasticity and poses problems for the reliable identification and classification of emerging pathogens, a necessary pre-requisite for using molecular surveillance as a tool for guiding effective use of existing antibiotics. For many years microbiological and molecular epidemiological surveillance studies within the veterinary and hospital environments have focused on characterizing their own targeted bacteria and antibiotic resistance profiles rather than taking a larger “one health” view of microbial resistance. Consequently, the molecular structures and CRL scaffolds that laterally mobilize drug resistance determinants between communities have been relatively under-studied. The sequences of plasmids; transposons; integrons insertion elements; genomic islands and other mobile elements need to be comprehended fully to unravel the real complexity of the problem. MDR evolves where antibiotics are used heavily, irrespective of whether it is in human clinical environments, food animal production settings, aquaculture or horticulture. Genetic phenomena like LGT inextricably links genes from disparate reservoirs to form CRL. These concepts, although increasingly recognized by the scientific community are not clearly communicated to the general public. Raised awareness of the complexity and far reaching implications associated with antibiotic resistance and a “one health” approach are critical for a global, sustained effort needed to alleviate the serious threat posed by multiple antibiotic resistant infectious agents. Molecular surveillance strategies embracing a “one health” approach are expected to provide a better understanding of how the genes flow through microbial communities and therefore provide a platform to more accurately predict and contain imminent threats posed by MDR bacteria.

One of the major challenges going forward is to stem the use of antibiotics and seek effective alternatives in our food production industries because antibiotic resistance gene reservoirs associated with food animals contribute to the evolution of MDR bacteria. One starting point for this is the development of alternatives to in-feed AGPs. This development should include studies that incorporate a full range of animals within the farming industry, as intestinal microbiomes are likely to differ between species. It should further include new approaches to gauge the effects of probiotics and alternate in-feed growth promoters.

### Conflict of interest statement

The authors declare that the research was conducted in the absence of any commercial or financial relationships that could be construed as a potential conflict of interest.
