# Supplementary material for: Genomic interplay in bacterial communities: implications for growth promoting practices in animal husbandry
Source: Front Microbiol. 2014 Aug 12;5:394. doi: 10.3389/fmicb.2014.00394 (PMC4129626; doi:10.3389/fmicb.2014.00394)
Supplement: Supplementary file 1 [file DataSheet1.DOCX]

| **AGP Alternative** | **Reference** | **Animal Target** | **Outcomes Observed** |
| --- | --- | --- | --- |
| Phytogenic compounds (botanicals, plant extracts) | Schöne *et al*. 2006 | Weaned piglets | Group fed diet supplemented with fennel oil showed no difference to control group (no additives), group fed diet supplemented with caraway oil showed less feed consumption and 10% less weight gain than control group. |
|  | Muhl and Liebert 2007 | Weaned piglets | Additive had no effect on growth, feed consumption, feed conversion rate or faecal microbial counts. |
|  | Ragland *et al.* 2007 | Nursery-age piglets | Diets supplemented with oregano oil resulted in decreased feed utilisation and average daily weight gain than control (no additives) and carbadox (antibiotic growth promoter) supplemented groups. |
| Neutraceuticals | Schöne *et al*. 2006 | Weaned piglets | Group fed diet supplemented with copper and formic acid showed 27% increase in feed consumption and 25% increase in weight gain. |
|  | Hill *et al.* 2000 | Weanling pigs | Pigs receiving feed supplemented with high levels (pharmacological concentrations) of copper and zinc gained weight quicker than those on the control (additive free) diet. Copper and zinc also increased feed intake. |
| Amino acids | Muhl and Liebert 2007 | Weaned piglets | Lysine supplementation did not influence faecal microbial counts but showed a significant increase in growth performance. |
|  | Askbrant *et al.* 1994 | Growing-finishing pigs | Greater growth rate and feed conversion ratios were observed in diets containing 13.5% crude protein supplemented with lysine, methionine and threonine, as opposed to an unsupplemented 15.8% crude protein diet. |
| Enzymes | Jacela *et al.* 2009 | Growing pigs | Addition of enzyme resulted in a lower average daily weight gain from days 0 to 28 compared to the control group (no enzyme), but increased from days 28 to 56. No overall difference was observed in growth performance between enzyme supplemented and control groups over the 56 day period. |
|  | Nortey *et al.* 2007 | Piglets under 6 months | Both xylase and phytase supplemented diets reduced feed intake and neither enzyme affected average daily gain. Xylase improved feed:gain ratio while phytase had no effect. Enzymes did not interact. |
|  | Barrerra *et al.* 2004 | Piglets (unspecified age) | The diet supplemented with xylanase resulted in a minute improvement with respect to ileal digestibility, average daily gain and feed:gain ratio. |
